# Effects of dietary curcumin supplementation on growth performance, intestinal morphology, and antioxidant status in weaned piglets: a meta-analysis

**DOI:** 10.3389/fvets.2026.1922106

**Published:** 2026-09-15

**Authors:** Wanrong Song, Zhengwei Chen, Yiduo Liu, Yifei Yang, Haiyan Wu, Ping Hu

**Affiliations:** 1 Department of Animal Science and Technology, Beijing Vocational College of Agriculture, Beijing, China; 2 Peking Union Medical College Hospital, Beijing, China; 3 Integrative Laboratory of Changzhou TCM Hospital Affiliated to Nanjing University of Chinese Medicine, Changzhou, China; 4 College of Animal Science, Fujian Agriculture and Forestry University, Fuzhou, China

**Keywords:** antioxidant status, curcumin, growth performance, intestinal morphology, meta-analysis, weaned piglets

## Abstract

Curcumin has attracted increasing attention as a natural feed additive with potential benefits for weaned piglets. This meta-analysis quantitatively evaluated its effects on growth performance, antioxidant status, and intestinal morphology. A total of 22 reports identified from PubMed, Web of Science, Embase, Scopus, Wiley Online Library, and CNKI were included in the quantitative synthesis. The main outcomes were average daily gain (ADG), average daily feed intake (ADFI), feed conversion ratio (FCR), villus height (VH), crypt depth (CD), villus height-to-crypt depth ratio (VH/CD), and antioxidant indices in serum and liver. Dietary curcumin supplementation showed beneficial effects on several growth, intestinal morphology, and antioxidant outcomes in weaned piglets. Curcumin supplementation increased ADG (SMD = 0.86, 95% CI: 0.55 to 1.17) and reduced FCR (MD = −0.75, 95% CI: −1.16 to −0.34), whereas its effect on ADFI was less consistent (SMD = 0.49, 95% CI: −0.00 to 0.98). Curcumin supplementation increased VH (SMD = 0.90, 95% CI: 0.57 to 1.22) and VH/CD (SMD = 1.04, 95% CI: 0.71 to 1.38), while reducing CD (SMD = −0.59, 95% CI: −0.85 to −0.33). Antioxidant effects were more consistent in the liver, with increased hepatic SOD (SMD = 1.20, 95% CI: 0.45 to 1.94), GSH-Px (SMD = 1.48, 95% CI: 0.28 to 2.68), and T-AOC (SMD = 1.49, 95% CI: 0.92 to 2.05), together with reduced hepatic MDA (SMD = −2.65, 95% CI: −3.37 to −1.93). Overall, curcumin showed beneficial effects on growth, redox status, and intestinal structure in weaned piglets, and its growth-promoting role appeared to be more closely associated with a reduction in FCR and post-weaning adaptation than with a stable increase in feed intake.

**Systematic review registration:**

https://doi.org/10.37766/inplasy2026.4.0054, identifier INPLASY (registration number INPLASY202640054).

## 1 Introduction

Weaning is one of the most critical and stress-intensive stages in pig production. The abrupt withdrawal of maternal nutritional and immune support, the transition from a liquid to a solid diet, and concurrent changes in the environment and social hierarchy collectively predispose piglets to reduced feed intake, impaired growth, a higher incidence of diarrhea, and disruption of intestinal function (1–3). Weaning is also frequently accompanied by villus atrophy, crypt hyperplasia, increased intestinal permeability, and oxidative stress, all of which may compromise nutrient digestion and absorption and ultimately impair subsequent health and productive performance (4–6).

In modern animal production, in-feed antibiotics were historically used to alleviate weaning-associated stress and improve growth performance. However, concerns regarding antimicrobial resistance, drug residues, and ecological safety have accelerated the search for effective and sustainable alternatives. Among the candidate strategies, phytogenic bioactive compounds have received considerable attention because of their broad biological activities and potential application as functional feed additives (7–9). Curcumin, a natural polyphenolic compound derived from turmeric, exhibits antioxidant, anti-inflammatory, free radical-scavenging, and immunomodulatory properties, and has therefore been regarded as a promising dietary additive for improving intestinal health and stress resilience in livestock (10, 11).

An increasing number of studies have investigated the effects of dietary curcumin supplementation in weaned piglets. Available evidence suggests that curcumin may contribute to improved growth performance, intestinal structure, and oxidative status during the post-weaning period (12, 13). In addition, turmeric-derived interventions have also been associated with favorable changes in gut microbial ecology and related physiological outcomes (14). However, the reported findings remain fragmented and, in some cases, inconsistent.

Piglets with intrauterine growth restriction (IUGR) represent a physiologically vulnerable subgroup characterized by impaired postnatal growth, intestinal development, metabolic homeostasis, and oxidative balance (15, 16). Previous evidence suggests that curcumin may exert antioxidant and intestinal effects in IUGR models (17–19). However, quantitative evidence specifically addressing weaned IUGR piglets remains limited. Therefore, health status was considered as one of the exploratory study characteristics in the present meta-analysis.

Substantial heterogeneity also exists across available studies. Differences in baseline piglet status, breed, curcumin source and formulation, supplementation level, intervention duration, and outcome selection may all contribute to the inconsistency of reported findings. Meta-analysis provides a more rigorous framework for quantitatively integrating dispersed evidence, estimating pooled effect sizes, and exploring potential sources of between-study heterogeneity.

Therefore, the present study conducted a meta-analysis to evaluate the effects of dietary curcumin supplementation on growth performance, intestinal morphology, and antioxidant status in weaned piglets. Subgroup analyses were further performed to explore whether the effects of curcumin varied according to baseline piglet status and other study characteristics.

## 2 Materials and methods

The protocol for this meta-analysis was registered with INPLASY (registration number: INPLASY202640054) and is available at: https://doi.org/10.37766/inplasy2026.4.0054.

### 2.1 Literature search

The literature search and study selection were conducted in accordance with the Preferred Reporting Items for Systematic Reviews and Meta-Analyses (PRISMA) guidelines. A systematic search was performed in PubMed, Web of Science, Embase, Scopus, Wiley Online Library, and China National Knowledge Infrastructure (CNKI) for eligible studies published in English or Chinese from database inception to March 1, 2026. To maximize the retrieval of relevant studies, combinations of Medical Subject Headings (MeSH), free-text terms, and Boolean operators were used. The main search terms included: (Curcumin Phytosome OR Phytosome, Curcumin OR Diferuloylmethane OR Turmeric Yellow OR Yellow, Turmeric OR Mervia OR Curcumin) AND (weanling pig OR weaned pig OR piglet). In addition, the reference lists of all eligible articles and relevant reviews were manually screened to identify any additional studies that may have been missed during the electronic search.

### 2.2 Inclusion and exclusion criteria

Studies were included in the meta-analysis if they met all of the following criteria: (1) the study population consisted of weaned piglets, including either healthy weaned piglets or piglets with IUGR; (2) the intervention involved dietary supplementation with curcumin or curcumin-related preparations; (3) the study included a control group fed a basal diet without curcumin supplementation; (4) at least one outcome of interest was reported, including growth performance, intestinal morphology, or antioxidant-related indices; (5) the study was a randomized controlled trial.

Studies were excluded if they met any of the following criteria: (1) the animals were not weaned piglets; (2) curcumin was administered in combination with other functional additives and the independent effect of curcumin could not be distinguished; (3) the study lacked a control group; (4) the outcomes of interest were not reported; (5) data were insufficient for quantitative synthesis and could not be obtained or converted; or (6) the publication was a review, conference abstract, book chapter, duplicate publication, or other non-original study.

When multiple publications reported overlapping data from the same experiment, only the most complete or most informative dataset was retained. No restrictions were imposed on pig breed, sex, supplementation dose, or intervention duration.

### 2.3 Data extraction

Data extraction was performed for all eligible studies using a standardized data collection form. The extracted study characteristics included first author and year, health status and model, breed, initial age and body weight, sample size, supplementation period, dose (mg/kg feed), and curcumin type and purity.

The extracted outcomes were categorized into three groups: growth performance, intestinal morphology and antioxidant status. Growth performance variables included average daily gain (ADG), average daily feed intake (ADFI), and feed conversion ratio (FCR). Intestinal morphology variables included villus height (VH), crypt depth (CD), and the villus height-to-crypt depth ratio (VH/CD). Antioxidant-related indices included superoxide dismutase (SOD), glutathione peroxidase (GSH-Px), total antioxidant capacity (T-AOC), and malondialdehyde (MDA).

For each outcome, the mean value, sample size, and corresponding measure of variance were extracted for both the curcumin-supplemented group and the control group. When data were reported as standard error (SE), they were converted to standard deviation (SD) using the formula SD = SE × √n. If outcomes were reported for different intestinal segments or tissues, the corresponding data were extracted separately where appropriate. When multiple curcumin doses were compared with a single control group, each eligible comparison was extracted separately. If numerical data were presented only in graphs, values were obtained from the figures whenever possible.

To ensure comparability across studies, units were standardized before quantitative synthesis when necessary. All extracted data were checked carefully prior to analysis, and any discrepancies in data coding or interpretation were resolved through repeated review of the original articles.

### 2.4 Statistical analysis

All statistical analyses were performed using Stata software (StataCorp, College Station, TX, USA). Meta-analyses were conducted to estimate the pooled effects of dietary curcumin supplementation on growth performance, intestinal morphology, and antioxidant status in weaned piglets.

For continuous outcomes, effect sizes were expressed as mean difference (MD) or standardized mean difference (SMD) with corresponding 95% confidence intervals (CIs), depending on the consistency of measurement scales across studies. Because most outcomes were measured using different units, tissues, or analytical methods, SMD was used in the majority of analyses.

Between-study heterogeneity was assessed using Cochran’s Q test and quantified with the *I^2^* statistic. *I^2^* values of 25, 50, and 75% were considered to represent low, moderate, and high heterogeneity, respectively. Given the expected variability among studies in terms of piglet health status, curcumin formulation, supplementation dose, and intervention duration, a random-effects model was applied for all analyses. Comparison-specific weights were calculated using the inverse-variance method. Under the random-effects model, the weight assigned to each comparison was calculated as follows:


$$w_{i}=1∕(v_{i}+\tau^{2})$$

where v_i_ represents the within-study sampling variance of the effect estimate and τ^2^ represents the estimated between-study variance.

Subgroup analyses were conducted for the main growth performance outcomes, including ADG, ADFI, and FCR. The predefined subgroup variables included health status, formulation, dose, and supplementation period. Health status was categorized as healthy piglets or intrauterine growth-restricted piglets. Formulation was grouped according to the type of curcumin-related preparation used in each study. Dose and supplementation period were categorized based on the distribution of the included data. These analyses were performed to explore potential sources of heterogeneity. A two-sided *p* value < 0.05 was considered statistically significant. Funnel plots were constructed to explore potential small-study effects regarding growth performance, intestinal morphology, and hepatic antioxidant outcomes. The robustness of the pooled estimates was further assessed using leave-one-out sensitivity analyses under the random-effects model, with each individual comparison sequentially omitted.

## 3 Results

The study selection process is shown in Figure 1. A total of 193 records were identified through database searching, and no additional records were identified through other sources. After removal of 78 duplicates, 115 records were screened by title and abstract, of which 59 were excluded. The remaining 56 full-text articles were assessed for eligibility, and 34 were further excluded because of absence of outcomes of interest, no control group, use of curcumin as part of a multi-component intervention, or inclusion of experimental animals other than weaned piglets. Finally, 22 reports were included in the quantitative synthesis. Because some included reports originated from the same experiment but presented different outcomes or treatment comparisons, these 22 reports were subsequently grouped into 14 independent studies for the purpose of summarizing baseline characteristics.

**Figure 1:**
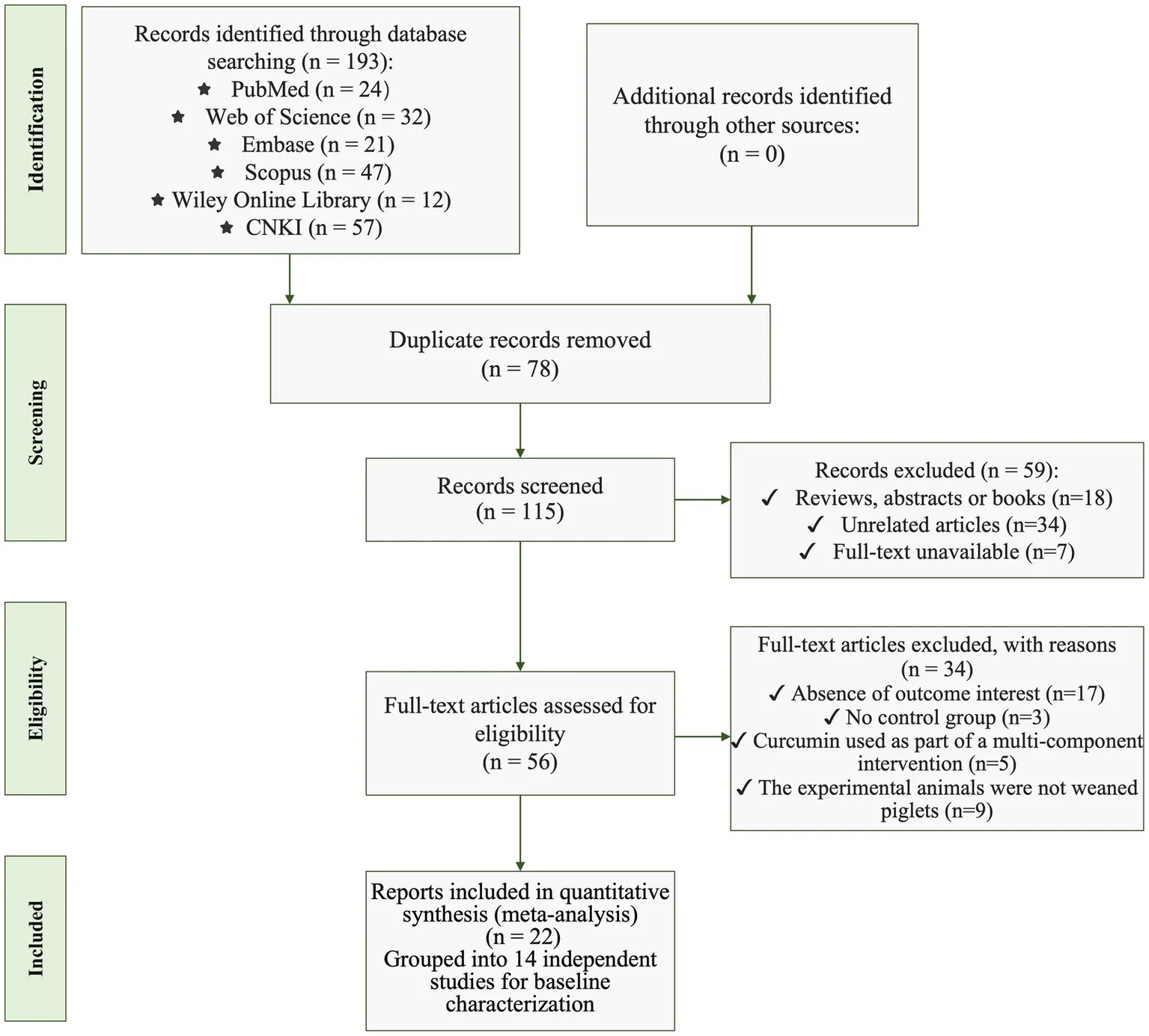
PRISMA flowchart for literature retrieval and research screening.

### 3.1 Characteristics of the included studies

A total of 22 reports met the eligibility criteria and were included in the quantitative synthesis. As some reports originated from the same experiment but provided different outcome data or treatment comparisons, they were grouped into 14 independent studies when summarizing the baseline characteristics. The baseline characteristics of these studies are presented in Table 1.

**Table 1:** Baseline characteristics of the studies included in the systematic review and meta-analysis.

First author and year	Health status	Breed	Initial age and body weight	Sample size	Supplementation period	Dose (mg/kg feed)	Curcumin type
Shi 2020 (12)	Healthy weaned piglets	Wuzhishan pig	Weaned at 35 days; 3.54 ± 0.28 kg	CON: *n* = 10 CUR: *n* = 10 (low)/*n* = 10 (high)	21 days	CUR: 200, 300	Curcumin
Recharla 2021 (14)	Healthy weaned piglets	Duroc × Landrace × Yorkshire	Weaned at 28 days; 7.35 ± 0.3 kg	CON: *n* = 24 CUR: *n* = 24	42 days	CUR: 1% w/w (~10,000)	Turmeric root powder
Moniruzzaman 2021 (20)	Healthy weaned piglets	Duroc × Large White × Landrace	28 ± 1 days; 7.0 ± 1.0 kg	CON: *n* = 45 CUR: *n* = 45 (low)/*n* = 45 (high)	21 days	CUR: 0.5 mL/kg, 1.0 mL/kg	Curcumin nanospheres
Ilsley 2005 (38)	Healthy weaned piglets	Large White × Landrace × Duroc	29 ± 0.1 days; 7.7 kg	CON: *n* = 48 CUR: *n* = 48	20 days	CUR: 200	Curcumin
Gan 2019a/b (23, 24); Wei 2020 (25)	Healthy weaned piglets	Duroc × Landrace × Yorkshire	28 ± 2 days; 7.8 ± 0.6 kg	CON: *n* = 30 CUR: *n* = 30	28 days	CUR: 300	Curcumin
Niu 2019a/b (18, 39), Wang 2019a/b (40, 41)	NBW/IUGR	Landrace × Yorkshire/Duroc × Landrace × Yorkshire	Weaned at 26 days; NBW: ~6.96 kg/IUGR: ~6.02 kg	NBW-CON: *n* = 8/10 NBW-CUR: *n* = 8/10 IUGR-CON: *n* = 8/10 IUGR-CUR: *n* = 8/10	24 days	CUR: 400	Curcumin
Yan 2020 (42), Zhang 2020/2022 (43, 44), Liu 2022 (45)	NBW/IUGR	Duroc × Landrace × Yorkshire	Weaned at 26 days; NBW: 7.52 kg/IUGR: 5.76 kg	NBW-CON: *n* = 12 IUGR-CON: *n* = 12 IUGR-CUR: *n* = 12	89–169 days	CUR: 200	Curcumin
Lu 2017 (46)	Healthy weaned piglets	Duroc × Landrace × Yorkshire	Weaned at 28 days; initial BW not specifically reported	CON: *n* = 75 CUR: *n* = 75 each (4 dose groups in total)	28 days	CUR: 100, 200, 300, 400	Curcumin
Deng 2018 (47)	Healthy piglets (growing stage)	Chuanzang Black pig	Weaning age not reported; ~15 kg	Antibiotic-free CON: *n* = 20 Synthetic CUR: *n* = 20 Natural CUR: *n* = 20	30 days	CUR: 500 (synthetic), 50 (natural)	Synthetic curcumin (purity 10%)/natural extract (purity 90%)
Xun 2016 (48)	Healthy weaned piglets	Duroc × Landrace × Yorkshire	Weaned at 21 days; initial BW not specifically reported	CON: *n* = 10 CUR: *n* = 10 each (3 dose groups in total)	21 days	CUR: 200, 300, 400	Curcumin
Wang 2022 (49)	Healthy weaned piglets	Duroc × Landrace × Yorkshire	Weaned at 24 days; 6.5–7.5 kg	CON: *n* = 12 CUR: *n* = 12 each (2 dose groups in total)	15 days	CUR: 200, 400	Curcumin
Hong 2022 (50)	Healthy weaned piglets	Duroc × Landrace × Yorkshire	Weaned at 28 days; 7.55 ± 0.42 kg	CON: *n* = 30 CUR: *n* = 30	14 days	CUR: 200	Curcumin
Li Binru 2024 (51)	Healthy weaned piglets	Duroc × Landrace × Yorkshire	Weaned at 28 days; 7.47 ± 0.58 kg	CON: *n* = 160 CUR: *n* = 160 each (3 dose groups in total)	42 days	CUR: 120, 240, 480	Turmeric extract (curcumin purity ≥95%)
Liu Hao 2024 (52)	Healthy weaned piglets	Duroc-Landrace-Yorkshire	Weaned at 26 days; 8.5 ± 0.5 kg	CON: *n* = 12 CUR: *n* = 12 each (3 dose groups in total)	35 days	CUR: 200, 400, 600	curcumin

(1) NBW, normal birth weight; IUGR, intrauterine growth restriction; BW, body weight; CUR, curcumin; CON, control. (2) Sample size (n) refers to the number of animals per group unless otherwise specified. (3) Studies labeled with the same first author and year followed by different letters [e.g., Niu (18, 39); Wang (40, 41)] indicate separate reports originating from the same experiment, usually presenting different outcomes or treatment comparisons. For baseline characterization, such reports were grouped as one independent study where appropriate.

### 3.2 Growth performance

A total of 23 comparisons were included for ADG (Figure 2A), 21 for ADFI (Figure 2B), and 21 for FCR (Figure 2C). Meta-analysis showed that dietary curcumin supplementation significantly increased ADG (SMD = 0.86, 95% CI: 0.55 to 1.17; *I^2^* = 18.7%, *P* for heterogeneity = 0.209) and significantly reduced FCR (MD = −0.75, 95% CI: −1.16 to −0.34; *I^2^* = 47.6%, *P* for heterogeneity < 0.01). For ADFI, the pooled estimate was (SMD = 0.49, 95% CI: −0.00 to 0.98), with substantial heterogeneity among studies (*I^2^* = 63.2%, *P* for heterogeneity < 0.001).

**Figure 2:**
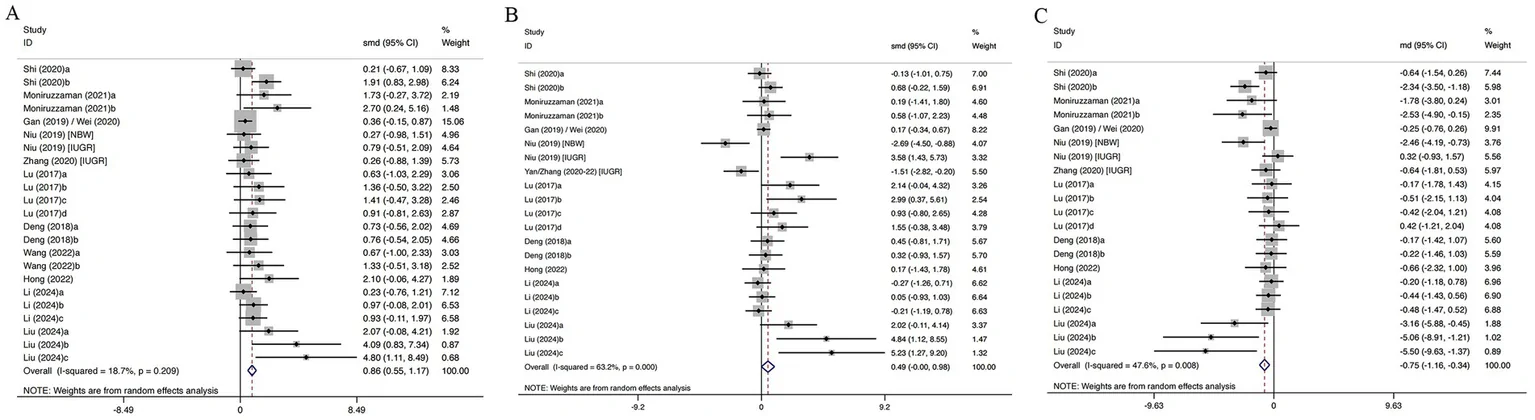
Forest plots of the effects of dietary curcumin supplementation on growth performance in weaned piglets: **(A)** Average daily gain (ADG), **(B)** Average daily feed intake (ADFI), and **(C)** Feed conversion ratio (FCR).

### 3.3 Intestinal morphology

Meta-analysis showed that dietary curcumin supplementation significantly increased VH (SMD = 0.90, 95% CI: 0.57 to 1.22; *I^2^* = 50.7%, *P* for heterogeneity < 0.01) (Figure 3A) and significantly increased VH/CD (SMD = 1.04, 95% CI: 0.71 to 1.38; *I^2^* = 52.6%, *P* for heterogeneity < 0.01) (Figure 3C). Curcumin supplementation also significantly decreased CD (SMD = −0.59, 95% CI: −0.85 to −0.33), with relatively lower heterogeneity among studies (*I^2^* = 30.5%, *P* for heterogeneity = 0.075) (Figure 3B). Overall, curcumin supplementation was associated with a favorable change in intestinal morphology, characterized by increased VH and VH/CD together with reduced CD.

**Figure 3:**
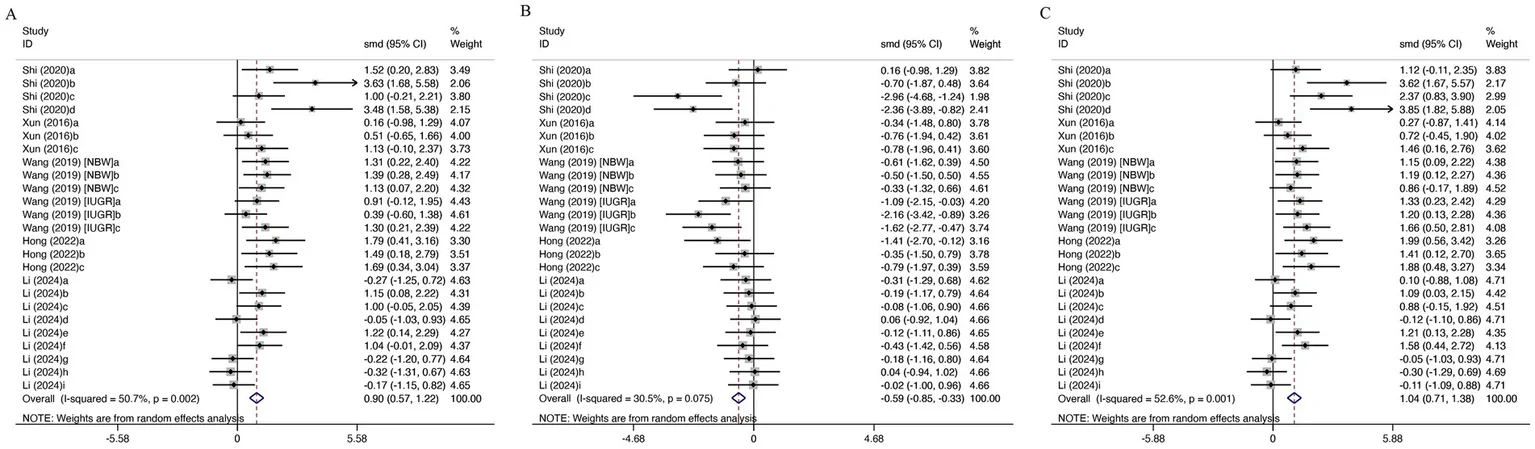
Forest plots of the effects of dietary curcumin supplementation on intestinal morphology in weaned piglets: **(A)** VH, **(B)** CD, and **(C)** VH/CD.

### 3.4 Antioxidant status

#### 3.4.1 Serum antioxidant indices

Meta-analysis of serum antioxidant indices showed generally favorable effects of dietary curcumin supplementation in weaned piglets. For serum SOD, the pooled analysis demonstrated a significant increase following curcumin supplementation (MD = 1.79, 95% CI: 0.66 to 2.92; *I^2^* = 69.4%, *P* for heterogeneity < 0.01) (Figure 4A). For serum GSH-Px, the pooled effect did not reach statistical significance (MD = 0.80, 95% CI: −0.38 to 1.97; *I^2^* = 80.8%, *P* for heterogeneity < 0.001) (Figure 4B). Likewise, no significant overall effect was observed for serum T-AOC (SMD = 0.56, 95% CI: −0.65 to 1.78; *I^2^* = 80.8%, *P* for heterogeneity < 0.001) (Figure 4C). In contrast, serum MDA was significantly reduced by curcumin supplementation (SMD = −1.10, 95% CI: −2.05 to −0.14; *I^2^* = 72.0%, *P* for heterogeneity < 0.001) (Figure 4D). Overall, the serum results were characterized by a significant increase in SOD activity and a significant reduction in MDA levels, whereas no significant changes were observed for GSH-Px or T-AOC.

**Figure 4:**
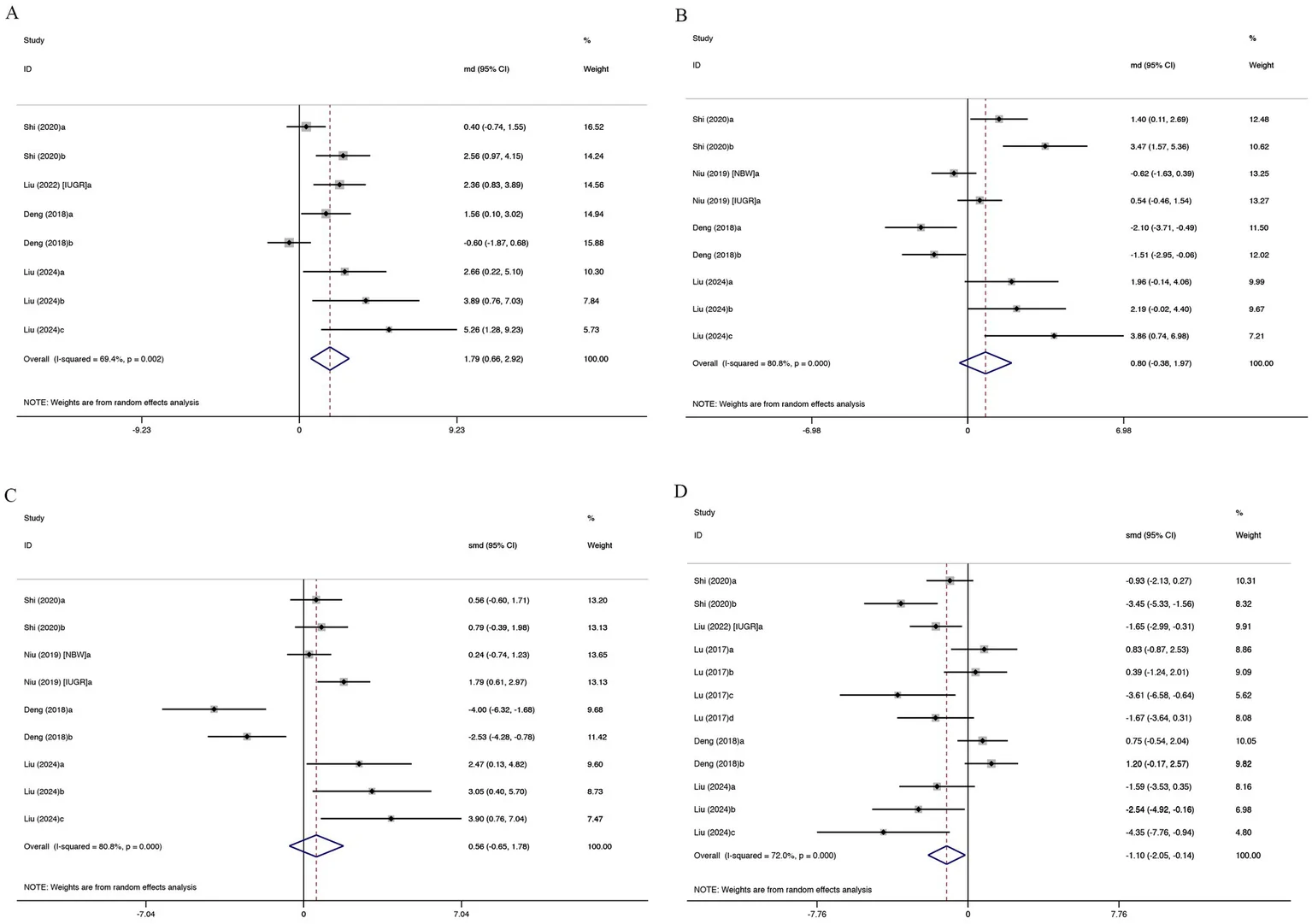
Forest plots of the effects of dietary curcumin supplementation on serum antioxidant indices in weaned piglets: **(A)** SOD, **(B)** GSH-Px, **(C)** T-AOC, and **(D)** MDA.

#### 3.4.2 Hepatic antioxidant indices

Compared with the serum results, the hepatic antioxidant indices showed a relatively consistent favorable pattern after curcumin supplementation. Hepatic SOD was significantly increased (SMD = 1.20, 95% CI: 0.45 to 1.94; *I^2^* = 78.2%, *P* for heterogeneity < 0.001) (Figure 5A). Hepatic GSH-Px was also significantly increased (SMD = 1.48, 95% CI: 0.28 to 2.68; *I^2^* = 89.5%, *P* for heterogeneity< 0.001) (Figure 5B). In addition, hepatic T-AOC was significantly elevated (SMD = 1.49, 95% CI: 0.92 to 2.05; *I^2^* = 62.1%, *P* for heterogeneity < 0.01) (Figure 5C). Hepatic MDA was significantly decreased (SMD = −2.65, 95% CI: −3.37 to −1.93; *I^2^* = 65.5%, *P* for heterogeneity< 0.001) (Figure 5D). Taken together, these findings support an overall favorable effect of curcumin supplementation on hepatic antioxidant capacity.

**Figure 5:**
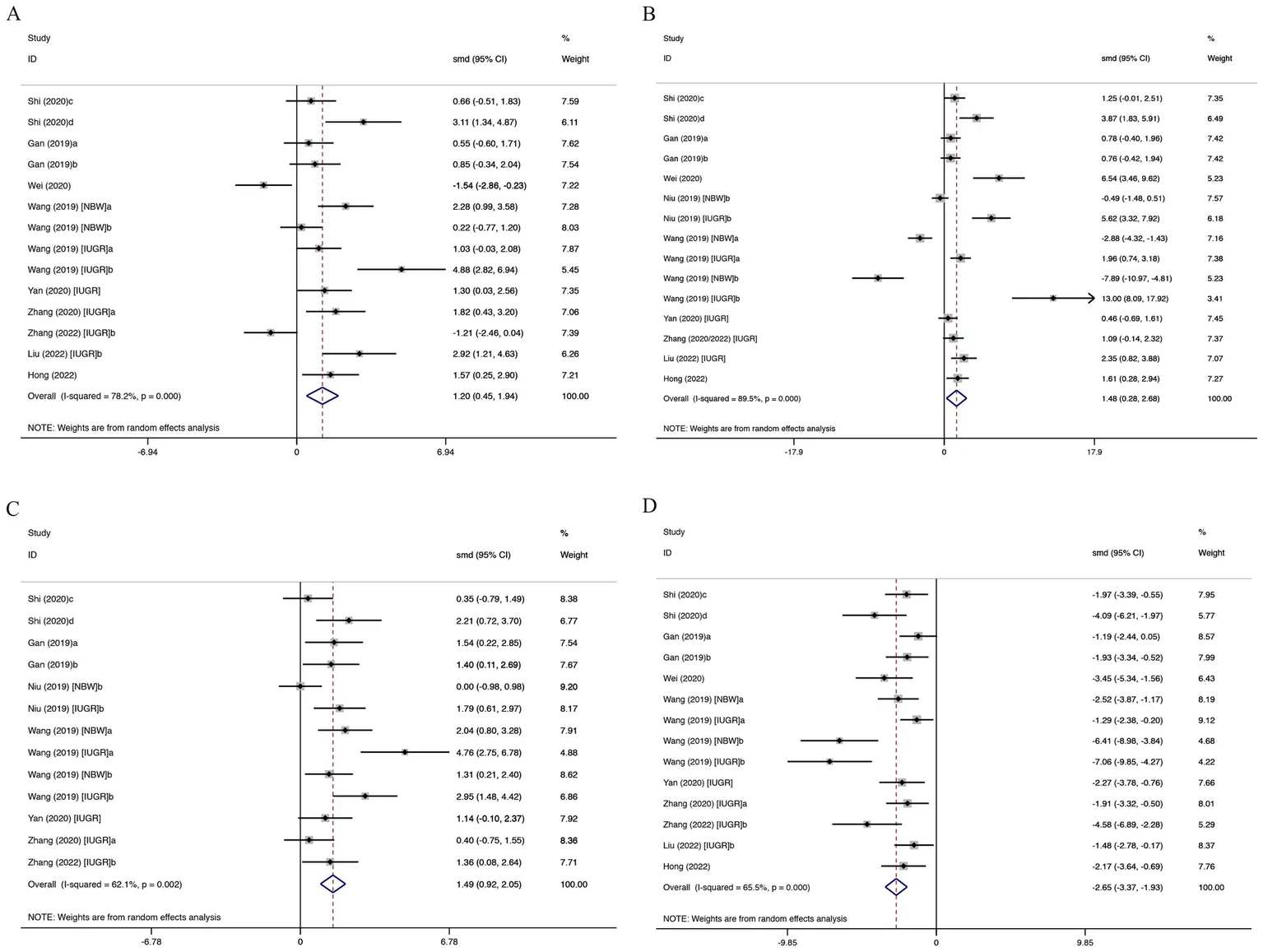
Forest plots of the effects of dietary curcumin supplementation on hepatic antioxidant indices in weaned piglets: **(A)** SOD, **(B)** GSH-Px, **(C)** T-AOC, and **(D)** MDA.

### 3.5 Subgroup analyses of growth performance

To further explore potential sources of heterogeneity and to evaluate whether the effects of dietary curcumin supplementation varied across study characteristics, subgroup analyses were performed for the three main growth performance outcomes, including ADG, ADFI, and FCR, according to health status, formulation, dose, and supplementation period. The results of these subgroup analyses are summarized in Table 2.

**Table 2:** Summary table of subgroup analysis results.

Subgroup variable	Subgroup	*n* (ADG/ADFI/FCR)	ADG			ADFI			FCR		
			Effect size (95% *CI*)	*I* ^2^	*p* (overall effect)	Effect size (95% *CI*)	*I* ^2^	*p* (overall effect)	Effect size (95% *CI*)	*I* ^2^	*p* (overall effect)
Health status	Healthy weaned piglets	20/18/18	0.97 (0.61, 1.33)	25.6%	*p* < 0.05	0.50 (0.10, 0.90)	38.6%	*p* < 0.05	−0.75 (−1.18, −0.31)	46.3%	*p* < 0.05
	NBW	1/1/1	0.27 (−0.98, 1.51)	—	ns	−2.69 (−4.50, −0.88)	—	*p* < 0.05	−2.46 (−4.19, −0.73)	—	*p* < 0.05
	IUGR	2/2/2	0.49 (−0.37, 1.34)	0.0%	ns	0.96 (−4.02, 5.94)	93.6%	ns	−0.19 (−1.13, 0.75)	16.9%	ns
Formulation	Conventional curcumin/curcumin extract	21/19/19	0.80 (0.49, 1.10)	15.6%	*p* < 0.05	0.52 (−0.02, 1.06)	66.8%	ns	−0.67 (−1.08, −0.26)	47.3%	*p* < 0.05
	Curcumin nanoparticles	2/2/2	2.11 (0.56, 3.66)	0.0%	*p* < 0.05	0.38 (−0.77, 1.53)	0.0%	ns	−2.09 (−3.63, −0.55)	0.0%	*p* < 0.05
Dose	Low dose: ≤200 mg/kg	9/8/8	0.57 (0.13, 1.01)	0.0%	*p* < 0.05	0.35 (−0.47, 1.16)	60.8%	ns	−0.52 (−0.97, −0.06)	0.0%	*p* < 0.05
	Moderate dose: 201–400 mg/kg	9/8/8	0.96 (0.44, 1.48)	35.1%	*p* < 0.05	0.70 (−0.23, 1.63)	75.4%	ns	−0.86 (−1.68, −0.04)	70.4%	*p* < 0.05
	High dose: >400 mg/kg	3/3/3	1.27 (−0.07, 2.61)	52.9%	ns	0.82 (−0.90, 2.55)	71.4%	ns	−0.94 (−2.52, 0.65)	66.0%	ns
Supplementation period	Short-term: ≤21 days	7/5/5	1.28 (0.55, 2.01)	35.2%	*p* < 0.05	0.28 (−0.24, 0.80)	0.0%	ns	−1.44 (−2.31, −0.57)	42.1%	*p* < 0.05
	Medium-term: 22–42 days	16/15/15	0.68 (0.37, 0.98)	3.6%	*p* < 0.05	0.81 (0.14, 1.48)	69.2%	*p* < 0.05	−0.52 (−0.97, −0.07)	41.5%	*p* < 0.05
	Long-term: >42 days	—/1/1	—	—	—	−1.51 (−2.82, −0.20)	—	*p* < 0.05	−0.64 (−1.81, 0.53)	—	ns

(1) n, number of individual comparisons for ADG/ADFI/FCR, respectively. (2) ns, not significant. (3) NBW, normal birth weight; IUGR, intrauterine growth restriction. (4) —, insufficient data or not applicable.

#### 3.5.1 ADG

In terms of health status, a significant increase in ADG was observed in healthy weaned piglets (SMD = 0.97, 95% CI: 0.61 to 1.33). In the NBW subgroup, the pooled estimate was SMD = 0.27 (95% CI: −0.98 to 1.51), although this result was based on only one comparison. In the IUGR subgroup, the pooled estimate was SMD = 0.49 (95% CI: −0.37 to 1.34). Although the point estimate was positive, only two comparisons were available and the confidence interval crossed the null value, indicating limited precision of the estimate. Nevertheless, the result provides preliminary quantitative information for this subgroup, for which pooled evidence remains limited (Figure 6A).

**Figure 6:**
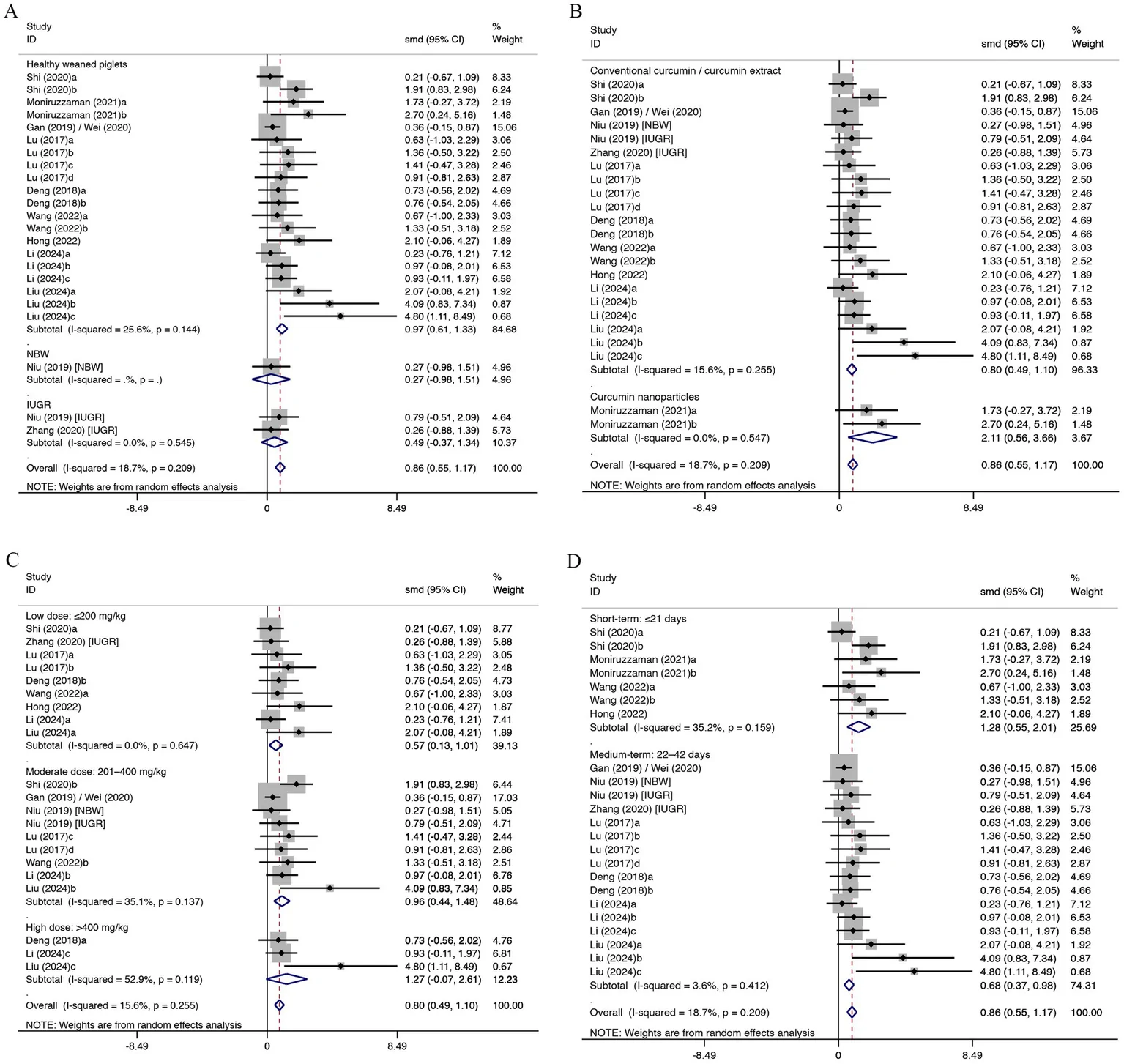
Forest plots of subgroup analyses for average daily gain (ADG): **(A)** health status, **(B)** formulation, **(C)** dose, and **(D)** supplementation period.

The formulation-based comparison showed a significant increase in ADG with conventional curcumin/curcumin extract (SMD = 0.80, 95% CI: 0.49 to 1.10) and with curcumin nanoparticles (SMD = 2.11, 95% CI: 0.56 to 3.66). The nanoparticle subgroup included fewer comparisons and had a wider confidence interval than the conventional formulation subgroup (Figure 6B).

The low-dose subgroup showed a significant increase in ADG (SMD = 0.57, 95% CI: 0.13 to 1.01), and the moderate-dose subgroup also showed a significant increase (SMD = 0.96, 95% CI: 0.44 to 1.48). The pooled estimate for the high-dose subgroup was SMD = 1.27 (95% CI: −0.07 to 2.61), with the confidence interval crossing the null value. These results did not indicate a clear dose–response relationship for ADG (Figure 6C).

Across supplementation periods, short-term supplementation (≤21 days) was associated with a significant increase in ADG (SMD = 1.28, 95% CI: 0.55 to 2.01), and the medium-term subgroup (22–42 days) also showed a significant increase (SMD = 0.68, 95% CI: 0.37 to 0.98). Evidence for the long-term subgroup (>42 days) was based on a limited number of comparisons. Overall, significant increases in ADG were observed in both the short- and medium-term subgroups, whereas the evidence for long-term supplementation remained limited (Figure 6D).

#### 3.5.2 ADFI

Compared with ADG, the subgroup results for ADFI were less consistent, which was in line with the weaker overall pooled effect. Looking first at health status, a more evident positive effect was observed in healthy weaned piglets. In contrast, the pooled effect in the normal-birth-weight (NBW) subgroup was negative (SMD = −2.69, 95% CI: −4.50 to −0.88). However, because this estimate was derived from only a single comparison, its stability is limited and it may be more appropriate to regard it as a suggestive result at present. In the IUGR subgroup, the pooled effect was positive in direction (SMD = 0.96, 95% CI: −4.02 to 5.94), but the wide confidence interval indicates considerable uncertainty. Overall, the current evidence for these two birth-weight-defined subgroups remains relatively weak, and further studies are needed to clarify their responses more reliably (Figure 7A).

**Figure 7:**
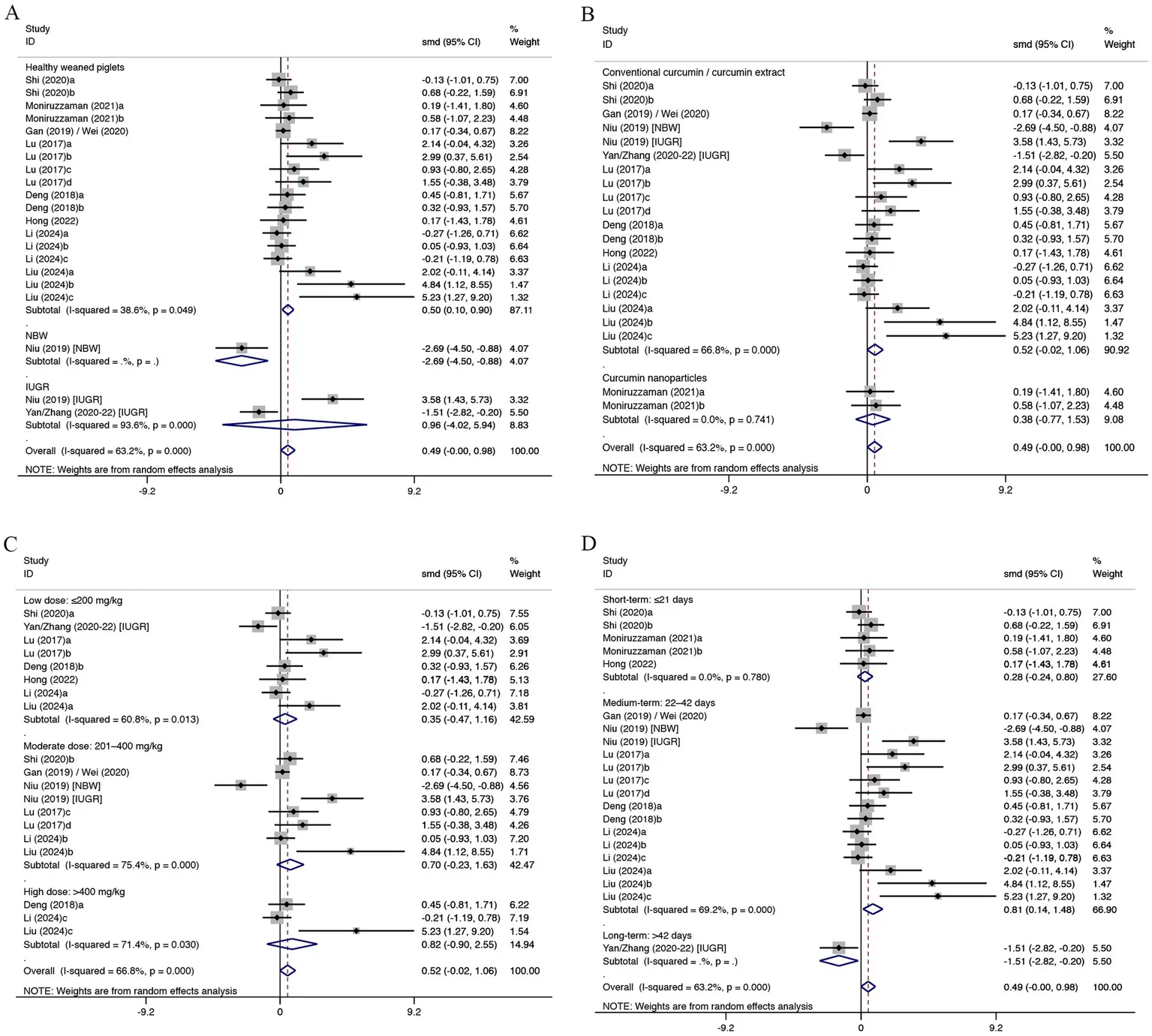
Forest plots of subgroup analyses for average daily feed intake (ADFI): **(A)** Health status, **(B)** Formulation, **(C)** Dose, and **(D)** Supplementation period.

The formulation-based analysis showed a pooled effect of SMD = 0.52 (95% CI: −0.02 to 1.06) for conventional curcumin/curcumin extract and SMD = 0.38 (95% CI: −0.77 to 1.53) for curcumin nanoparticles. The confidence intervals crossed the null value in both subgroups. Conventional preparations were represented by more comparisons than nanoparticle formulations. Overall, no statistically significant increase in ADFI was observed in either formulation subgroup, and the limited number of nanoparticle comparisons restricts the interpretation of the formulation-specific results (Figure 7B).

The pooled estimates were SMD = 0.35 (95% CI: −0.47 to 1.16) for the low-dose subgroup (≤200 mg/kg), SMD = 0.70 (95% CI: −0.23 to 1.63) for the moderate-dose subgroup (201–400 mg/kg), and SMD = 0.82 (95% CI: −0.90 to 2.55) for the high-dose subgroup (>400 mg/kg). The confidence intervals crossed the null value in all three dose subgroups, providing no evidence of a clear dose–response relationship for ADFI (Figure 7C).

Short-term supplementation (≤21 days) yielded an SMD of 0.28 (95% CI: −0.24 to 0.80), whereas medium-term supplementation (22–42 days) yielded an SMD of 0.81 (95% CI: 0.14 to 1.48). The confidence interval crossed the null value for the short-term subgroup but not for the medium-term subgroup. Evidence for the long-term subgroup (>42 days) was based on a limited number of comparisons. Overall, the available evidence supports an increase in ADFI during medium-term supplementation, whereas no clear effect was demonstrated for short-term supplementation and evidence for long-term supplementation remained limited (Figure 7D).

#### 3.5.3 FCR

Subgroup analyses indicated that the favorable effect of curcumin supplementation on FCR was generally maintained across most subgroup classifications, although the magnitude of the pooled effect varied. With regard to health status, the pooled estimate was negative in healthy weaned piglets (SMD = −0.75, 95% CI: −1.18 to −0.31), indicating a relatively consistent improvement in FCR in this subgroup. By contrast, the estimate in normal-birth-weight (NBW) piglets was also negative but was derived from only a single comparison (SMD = −2.46, 95% CI: −4.19 to −0.73), whereas the pooled effect in IUGR piglets remained less stable and was not statistically clear (SMD = −0.19, 95% CI: −1.13 to 0.75). Overall, these findings suggest that the reduction in FCR was more consistently observed in healthy weaned piglets, while the evidence in birth-weight-defined subgroups remains limited (Figure 8A).

**Figure 8:**
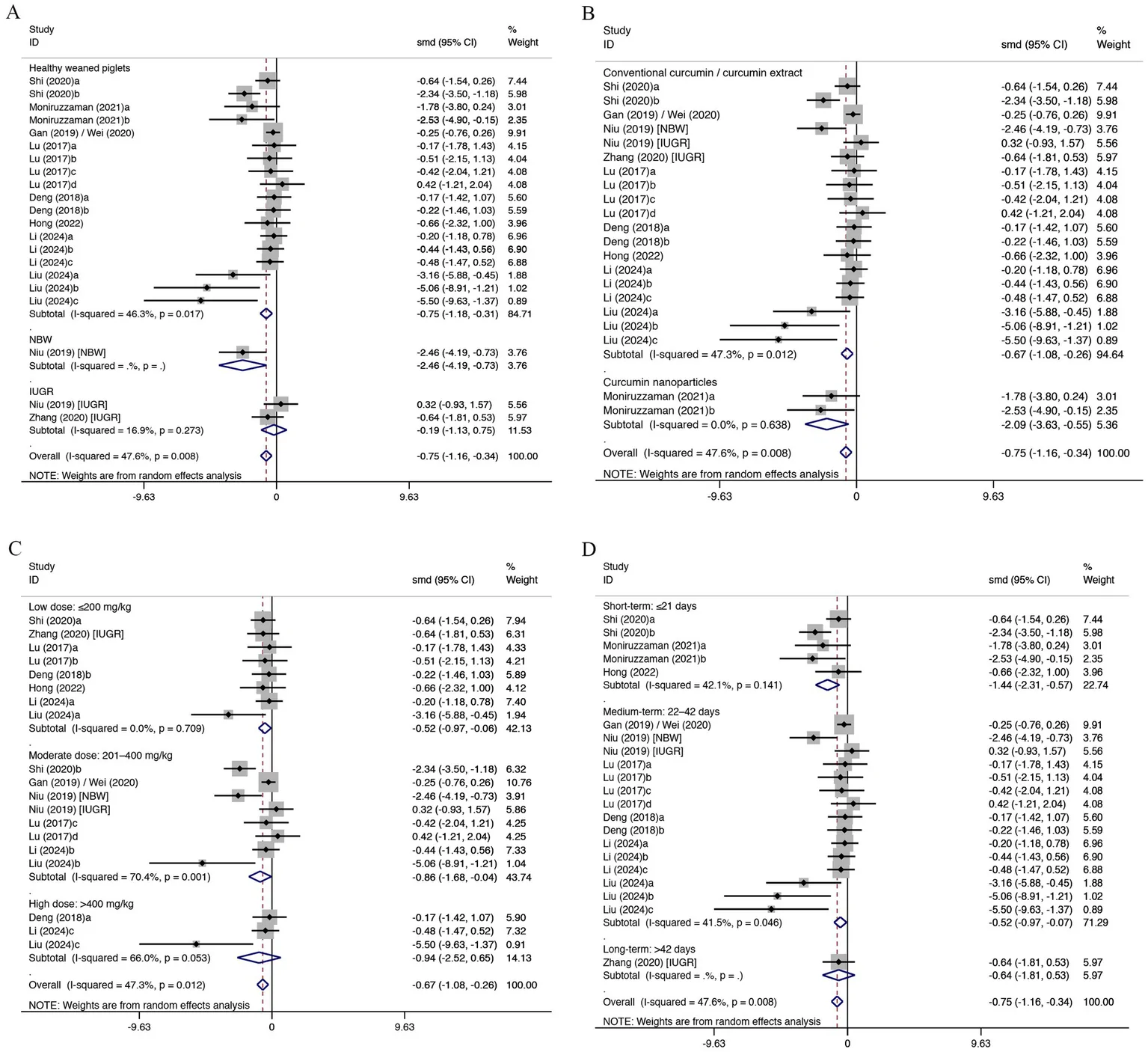
Forest plots of subgroup analyses for feed conversion ratio (FCR): **(A)** Health status, **(B)** Formulation, **(C)** Dose, and **(D)** Supplementation period.

The formulation-based analysis showed a significant reduction in FCR with conventional curcumin/curcumin extract (SMD = −0.67, 95% CI: −1.08 to −0.26) and with curcumin nanoparticles (SMD = −2.09, 95% CI: −3.63 to −0.55). The nanoparticle subgroup was based on fewer comparisons than the conventional formulation subgroup (Figure 8B).

Significant reductions in FCR were observed in the low-dose subgroup (≤200 mg/kg; SMD = −0.52, 95% CI: −0.97 to −0.06) and the moderate-dose subgroup (201–400 mg/kg; SMD = −0.86, 95% CI: −1.68 to −0.04). For the high-dose subgroup (>400 mg/kg), the pooled estimate was SMD = −0.94 (95% CI: −2.52 to 0.65), with the confidence interval crossing the null value. These results did not indicate a clear linear dose–response relationship for FCR (Figure 8C).

A broadly similar pattern was seen across supplementation periods. Short-term supplementation (≤21 days) showed a significant reduction in FCR (SMD = −1.44, 95% CI: −2.31 to −0.57), and the medium-term subgroup (22–42 days) also showed a significant negative pooled effect (SMD = −0.52, 95% CI: −0.97 to −0.07). In contrast, the long-term subgroup (>42 days) was represented by only one comparison and did not show a clear advantage (SMD = −0.64, 95% CI: −1.81 to 0.53). Taken together, these findings suggest that the favorable effect of curcumin supplementation on FCR can already be observed during short-term intervention and may remain present during the medium-term period, whereas long-term evidence is still insufficient to support a stable conclusion (Figure 8D).

### 3.6 Sensitivity analysis

Funnel plots showed that the individual comparisons were generally distributed on both sides of the pooled estimates, although some asymmetry was observed for certain outcomes (Appendix 1 in Supplementary material). In the leave-one-out analyses, the pooled estimates remained in the same direction and changed only modestly after each comparison was excluded. For a few outcomes, the 95% confidence interval occasionally crossed the null value after exclusion of a single comparison; however, no effect changed to the opposite direction (Appendix 2 in Supplementary material). Overall, no single comparison materially altered the overall pattern of the results.

## 4 Discussion

In this meta-analysis, dietary curcumin supplementation improved growth performance in weaned piglets, as shown by higher ADG and lower FCR, while the response of ADFI was relatively weak and less consistent. At the same time, curcumin was associated with more favorable intestinal morphology, including increased villus height and VH/CD ratio together with reduced crypt depth, and with an overall improvement in antioxidant status, particularly lower MDA and higher antioxidant enzyme activity. Viewed together, these findings support the idea that the main mechanism by which curcumin promotes post-weaning growth is through improving feed utilization and antioxidation, rather than continuously increasing feed intake (12, 14, 20).

The contrast between ADG/FCR and ADFI is particularly noteworthy. If curcumin acted mainly by stimulating appetite, a clearer and more stable increase in ADFI would have been expected. That was not what the pooled results showed. Instead, the overall pattern is closer to a scenario in which piglets made better use of the feed they consumed. A similar observation has been reported in housed growing lambs, where curcumin increased ADG without significantly affecting dry matter intake, while also improving ruminal fermentation, microbial protein synthesis, and antioxidant capacity (21, 22). Related pig studies using curcumin nanospheres also reported improved growth performance and feed utilization, whereas the response of feed intake was less clearly pronounced (20). This makes it more reasonable to view curcumin as a modulator of efficiency and adaptation rather than a simple intake-promoting additive.

One plausible explanation is that curcumin improves growth by preserving intestinal structure and function during the post-weaning period. During the post-weaning period, rapid changes in diet, environment, and immune status may temporarily affect villus architecture, epithelial turnover, and digestive and absorptive capacity as piglets adapt to the new conditions. Against that background, the present changes in VH, CD, and VH/CD are not just structural findings; they are closely related to the animal’s ability to extract nutrients from the diet. Several piglet studies are in line with this interpretation. Shi et al. (12) showed that curcumin improved villus morphology, enhanced tight-junction-related gene expression, and lowered markers of intestinal permeability in weaned piglets. Dietary turmeric supplementation has also been reported to improve growth together with a higher ileal VH/CD ratio, increased short-chain fatty acid production, and altered bacterial composition (14). Other studies further suggest that curcumin-containing interventions help preserve mucosal integrity and barrier-related function in weaning piglets (23, 24). Put simply, if the intestine is structurally more intact and functionally more stable, the animal may gain more weight from the same amount of feed.

This also helps explain why ADG increased more clearly than ADFI. Growth after weaning is not determined by intake alone. It also depends on how much of the ingested nutrients can actually be digested, absorbed, and directed toward tissue accretion. Some piglet studies suggest that curcumin-based supplementation can improve digestive enzyme activity, mucosal function, and intestinal microbial ecology (25, 26). It suggests that curcumin may be improving the efficiency of nutrient handling after ingestion.

Oxidative stress appears to be another important part of the explanation. Weaning is not merely a biochemical disturbance. It also increases the metabolic burden of maintaining intestinal and systemic homeostasis. This burden can be even greater in IUGR piglets. In the current analysis, curcumin generally lowered MDA and increased antioxidant defense, especially in hepatic outcomes. Previous pig studies provide mechanistic support for this. In IUGR piglets, curcumin increased antioxidant enzyme activity and upregulated Nrf2- and Hmox1-related signaling, while in growing pigs it alleviated jejunal injury through the Nrf2/Keap1 pathway (18). Other work has shown that curcumin can protect against oxidative-stress-induced intestinal damage by improving mitochondrial function, reducing endoplasmic reticulum stress, and restoring intracellular homeostasis (27–29). These findings suggest that the antioxidant role of curcumin is broader than direct free-radical scavenging alone and may involve regulation of stress-response pathways at both tissue and cellular levels. This mechanistic background also provides a biological rationale for exploring IUGR as a distinct subgroup in the present meta-analysis. Although the limited comparative data currently available preclude definitive conclusions regarding the IUGR subgroup, the preliminary quantitative evidence derived from this analysis offers a reference for future research on IUGR. From this perspective, the increase in ADG and reduction in FCR observed in the present analysis may reflect not only better digestion and absorption, but also a lower physiological burden associated with oxidative stress.

Findings from other animal species point in the same general direction. In ducks, curcumin supplementation improved growth performance together with antioxidant capacity (30, 31). In broilers, curcumin has been linked to better growth, improved intestinal integrity, and reduced inflammatory responses, with modulation of gut microbiota and microbial metabolites proposed as part of the mechanism. Similar observations have been made in slow-growing chickens under stressful conditions, where curcumin-containing nanoformulations improved growth performance, intestinal morphology, and oxidative status even when feed intake was not consistently changed (32–34). Although species differences must be considered, these studies support the interpretation that curcumin may improve the physiological conditions that support growth rather than simply increasing feed consumption.

The subgroup results also showed variation across different study conditions. However, the NBW- and IUGR-defined subgroups were represented by very few comparisons. Nevertheless, these analyses provide preliminary quantitative information for a biologically relevant subgroup and help identify an important evidence gap for future targeted studies. The same caution applies to nanoparticle formulations and longer supplementation periods. Likewise, the dose-related results did not show a simple linear pattern, which suggests that higher inclusion levels are not automatically associated with greater benefit. This is not unusual for curcumin, since response size is likely shaped by formulation, bioavailability, physiological background, and the type of challenge being imposed (35–37).

Several limitations should be considered when interpreting these findings. The included studies varied in animal model, formulation, dose, duration, and experimental context, all of which may have contributed to between-study variability. Some subgroup estimates were based on only a few comparisons.

Overall, the present findings support curcumin as a promising functional feed additive for weaned piglets. Its main value appears to lie less in increasing feed intake and more in helping piglets cope with the post-weaning transition. Better gut structure, lower oxidative burden, and improved physiological adaptation may contribute to that response. Future studies should pay more attention to formulation optimization, practical dose selection, and the biological pathways through which curcumin improves growth and health in both healthy and growth-restricted piglets.

## 5 Conclusion

Dietary curcumin supplementation showed overall beneficial effects in weaned piglets by improving growth performance, intestinal morphology, and antioxidant status. Curcumin increased average daily gain and reduced feed conversion ratio, whereas its effect on average daily feed intake was weaker and less consistent, suggesting that its growth-promoting role is more closely related to improved feed conversion ratio than to a stable increase in feed intake. Curcumin supplementation was also associated with increased villus height and villus height-to-crypt depth ratio, reduced crypt depth, enhanced antioxidant defense, and lower lipid peroxidation, with more consistent antioxidant responses observed in the liver than in serum. Overall, curcumin appears to be a promising nutritional strategy for supporting post-weaning adaptation in piglets. Further studies are needed to optimize formulation, dose, and supplementation duration and to clarify the mechanisms underlying these effects.

## Data Availability

The raw data supporting the conclusions of this article will be made available by the authors, without undue reservation.
