# Supplementary material for: Effects of dietary curcumin supplementation on growth performance, intestinal morphology, and antioxidant status in weaned piglets: a meta-analysis
Source: Front Vet Sci. 2026 Sep 15;13:1922106. doi: 10.3389/fvets.2026.1922106 (PMC13619526; doi:10.3389/fvets.2026.1922106)
Supplement: Supplementary file 1 [file Supplementary_file_1.DOCX]

**Appendix 1: Funnel plot of growth performance, intestinal morphology, and hepatic antioxidant outcomes**

**Appendix 2: Sensitivity analysis of ADG (A), ADFI (B), FCR (C), VH (D), CD (E), VH/CD (F), serum SOD (G), serum GSH-Px (H), serum T-AOC (I), serum MDA (J), Hepatic SOD (K), hepatic GSH-Px (L), hepatic T-AOC (M) and hepatic MDA (N).**

B

A

D

C

F

E

H

G

I

J

L

M

K

N
